# Executive Functions and Prosodic Abilities in Children With High-Functioning Autism

**DOI:** 10.3389/fpsyg.2018.00359

**Published:** 2018-03-21

**Authors:** Marisa G. Filipe, Sónia Frota, Selene G. Vicente

**Affiliations:** ^1^Center of Linguistics, School of Arts and Humanities, University of Lisbon, Lisbon, Portugal; ^2^Centre for Psychology, Faculty of Psychology and Education Sciences, University of Porto, Porto, Portugal

**Keywords:** executive functions, prosody, prosodic skills, high-functioning autism, autism spectrum disorders

## Abstract

Little is known about the relationship between prosodic abilities and executive function skills. As deficits in executive functions (EFs) and prosodic impairments are characteristics of autism, we examined how EFs are related to prosodic performance in children with high-functioning autism (HFA). Fifteen children with HFA (*M* = 7.4 years; *SD* = 1.12), matched to 15 typically developing peers on age, gender, and non-verbal intelligence participated in the study. The Profiling Elements of Prosody in Speech-Communication (PEPS-C) was used to assess prosodic performance. The Children’s Color Trails Test (CCTT-1, CCTT-2, and CCTT Interference Index) was used as an indicator of executive control abilities. Our findings suggest no relation between prosodic abilities and visual search and processing speed (assessed by CCTT-1), but a significant link between prosodic skills and divided attention, working memory/sequencing, set-switching, and inhibition (assessed by CCTT-2 and CCTT Interference Index). These findings may be of clinical relevance since difficulties in EFs and prosodic deficits are characteristic of many neurodevelopmental disorders. Future studies are needed to further investigate the nature of the relationship between impaired prosody and executive (dys)function.

## Introduction

There has been a recent interest in the study of the relationship between executive functions (EFs) and communication skills in typical and atypical development (e.g., Bishop and Norbury, 2005; Ellis Weismer et al., 2005; Im-Bolter et al., 2006; Henry et al., 2012; Vugs et al., 2014). In typical development, a link has been suggested between inhibition and lexical and syntactic disambiguation in children and young adults (Khanna and Boland, 2010). Working memory has been associated with auditory and written sentence comprehension in children and adults (e.g., Daneman and Carpenter, 1980; Roberts et al., 2007) and with sentence production in young adults (Slevc, 2011). In atypical development, difficulties in EFs have been observed in populations with communication impairments. For example, children with specific language impairment tend to have lower scores than typically developing (TD) peers on measures that assess EFs, including inhibition (Bishop and Norbury, 2005; Im-Bolter et al., 2006), task-shifting (Marton, 2008), and working memory (Ellis Weismer et al., 2005; Henry et al., 2012; Vugs et al., 2014). Deficits in EFs have also been observed in other disorders that include communication challenges, such as aphasia (Yeung and Law, 2010), traumatic brain injury (e.g., Sainson et al., 2014), and autism spectrum disorders (ASD) (e.g., Joseph et al., 2005). Crucially, EFs and language abilities seem to be related, both in comprehension and production, and prior findings suggest a link between communication impairments and deficits in EFs.

In line with evidence suggesting that EFs are closely related to language abilities, several models of language impairment now propose that language performance includes cognitive factors such as processing speed, attention, and EFs in addition to linguistic ability (Montgomery, 2002; Gomes et al., 2007; Leonard et al., 2007; Montgomery and Windsor, 2007; Bishop et al., 2014). Bishop et al. (2014) proposed three models to explain the relationship between EFs and language skills: (a) EFs influence the development of language; (b) children use verbal facilitation to assist them in EFs tasks; and (c) there is no causal relationship between these skills, and it is possible that shared problems in the development of the nervous system could account for the correlations. Gooch et al. (2016) described a further alternative: EFs and language skills may develop in a reciprocal interaction, and the relationship could change over time. In this context, longitudinal studies provided a starting point for the understanding of this relationship. Kuhn et al. (2014) studied the link between children’s early communicative gestures at 15 months, language abilities at 2/3 years, and EFs at 4 years of age, and they found that early language skills predicted later EFs. Exploring the relationship between language and EFs in children at-risk for language learning impairments in the transition from preschool to schooling, Gooch et al. (2016) found a strong concurrent relationship between language and EFs. Therefore, EFs and language performance are related, and theoretically this could also be true for prosodic performance.

Prosody plays an important role in communication disorders, as difficulties with prosodic skills can impact on language abilities in general and dramatically influence daily conversations, social interactions (Shriberg et al., 2001; Paul et al., 2005), and even typical language development (e.g., Cutler and Swinney, 1987; Frota et al., 2016). For example, prosody has been shown to play an important role in lexical and syntactic acquisition (Christophe et al., 2008; Hawthorne and Gerken, 2014; de Carvalho et al., 2016). Indeed, prosody is crucial for the production and comprehension of the organization of speech, manifested by patterns of intonation, rhythm, prominence, and chunking of the speech continuum (Wagner and Watson, 2010). Prosodic features impact not only on “*how we say it*” but also on “*what we say*.”

However, very little is known about the relationship between cognitive processes and prosodic abilities, and an important theoretical question is whether prosody is independent of other cognitive aspects such as EFs. Given that deficits in EFs and prosodic impairments are both characteristics of autism, this study investigates how EFs are related to prosodic performance in children with high-functioning autism (HFA), thus contributing to our understanding about the cognitive mechanisms that underlie language development.

## Autism Spectrum Disorders

Autism spectrum disorders (ASD) are a complex group of neurodevelopmental disorders, evident by early childhood, in which the severity of symptoms ranges from minor to incapacitating impairments. Common manifestations are repetitive or stereotyped interests, mannerisms, and difficulties in social communication (American Psychiatric Association, 2013). Regarding intellectual abilities, 44% of children affected with ASD are reported to have an average intellectual ability, 24% have a borderline intelligence quotient (IQ), and 32% have an intellectual disability (Christensen et al., 2016). The children without intellectual disabilities are often referred to as having HFA. Although autism is a disorder characterized by multiple impairments, including deficits in EFs and prosody, research has failed to clearly document the relationship between ASD, language, cognition, and EFs.

## Executive Functions in Autism Spectrum Disorders

Impairments in EFs have been considered a central deficit in autism (e.g., Rajendran and Mitchell, 2007), and investigating aspects of EFs in ASD has been an active area of research. Specifically, studies have indicated that children with ASD struggle with tasks requiring working memory, inhibition, and set-shifting abilities (e.g., Ozonoff et al., 1991, 2005; Hughes et al., 1994; Ozonoff and McEvoy, 1994; Ozonoff and Jensen, 1999; Adams and Jarrold, 2012). Additionally, a dysfunction frequently found is the perseveration of behavior, that is, the tendency to continue to perform actions that are no longer appropriate to the context (e.g., Rumsey and Hamburger, 1988; Prior and Hoffman, 1990). Children with ASD have also shown deficits in shifting attention, and in sustained or selective attention (Noterdaeme et al., 2001; Landry and Bryson, 2004). Furthermore, O’Hearn et al. (2008), in a review, reported that impairments in tasks requiring response inhibition, working memory, planning, and attention are also present in adulthood. In fact, impairments in EFs could be a potential explanation for many features of ADS, including difficulties with planning, inhibition, flexibility, and working memory.

## Prosodic Skills in Autism Spectrum Disorders

Prosodic impairments appeared amongst the first clinical descriptions of autism (Kanner, 1943; Asperger, 1944), and currently diagnostic tools of ASD include atypical expressive prosody as a feature of ASD (e.g., the Autism Diagnostic Interview-Revised, ADI-R, Lord et al., 1994; and the Autism Diagnostic Observation Schedule, ADOS, Lord et al., 1989).

Prosodic impairments in ASD have been extensively investigated from the viewpoint of perception and production. Deficits in the perception of prosodic features in individuals with ASD have been described, for example, in the comprehension of emphatic stress (Paul et al., 2005), as well as in the perception of pairs of the same auditory stimuli as prosodically different (Peppé et al., 2007). Impairments in expressive prosody in individuals with ASD have been described for rhythm, rate of speech, intonation patterns (e.g., Shriberg et al., 2001; McCann and Peppé, 2003; Paul et al., 2005), and the use of prosody to convey phrase-level prominence (McCann et al., 2007). However, some findings are controversial. For instance, monotone intonation has been reported but so has exaggerated intonation (Kanner, 1943; Baltaxe and Simmons, 1985; Sharda et al., 2010; Bonneh et al., 2011; DePape et al., 2012; Filipe et al., 2014), and slow syllabic speech has been described together with fast articulation rate (Baron-Cohen and Staunton, 1994; for a review, see McCann and Peppé, 2003).

In sum, results on prosody in ASD are mixed, with no agreement between studies. So far, no convincing explanation for these discrepant findings has been put forward. This atypical variation might be explained by methodological problems related to the assessment of prosody, poor diagnostic data, small sample sizes, and lack of appropriate comparison groups (e.g., McCann and Peppé, 2003; Diehl et al., 2009), but also by the multiplicity and heterogeneity of symptoms in ASD (Shriberg et al., 2001; Rice et al., 2005). Therefore, there is a current need for research in this field that takes in account the link between symptoms in ASD, such as cognitive abilities and prosodic skills.

## Present Study

This study examines EFs and prosody in children with HFA and TD peers. To the best of our knowledge, no study has yet analyzed the relation between EFs and prosodic skills, although evidence suggests that EFs are closely related to language abilities and that these are crucial foundations for development and learning. Since deficits in EFs and prosodic impairments may be a common feature of many disorders, including neurodevelopmental disorders such as ASD, we examined EFs performance and prosodic performance in HFA to determine whether prosodic abilities are associated with EFs, and if so to what extent and with what particular functions. Furthermore, we wanted to investigate if prosodic abilities are mediating differences in EFs performance, or if the reverse pattern was found. This specific clinical population offers methodological advantages because it separates out the confounding cognitive issues seen in other atypical populations. Specifically, the analyses aim to address the following research questions: (a) Does atypical development (i.e., HFA) affect performance on tests that assess prosodic skills and EFs?; (b) Do prosodic skills correlate with EFs measures in the HFA group?; and (c) Do prosodic skills mediate the differences in EFs between the HFA group and the TD group, or does the reverse pattern hold?

## Materials and Methods

### Participants

Fifteen children (3 girls, 12 boys) with HFA (6–9 years; *M* = 7.40, *SD* = 1.12), who met the DSM-5 criteria for Autism (American Psychiatric Association, 2013), participated in the study. A team of child-psychiatrists and psychologists made the diagnosis of ASD. The materials used in the diagnostic procedure were the Autism Diagnostic Interview-Revised (ADI-R; Lord et al., 1994) and the Autism Diagnostic Observation Schedule (ADOS; Lord et al., 1989). Participants characteristics are shown in **Table 1**. All HFA participants were required to have an IQ of 80 or higher (assessed with Wechsler Intelligence Scale for Children-III; Wechsler, 1991), to control for poor performance on the prosodic tasks not being a general consequence of cognitive impairments. Exclusion criteria were obsessive–compulsive disorders, attention deficit hyperactivity disorder, and learning disorders, according to DSM-5. The group with HFA was matched to a TD group on age (*M* = 7.53, *SD* = 0.99), gender, and non-verbal intelligence (HFA: *M* = 25.33, *SD* = 5.10; TD: *M* = 24, *SD* = 4.22; assessed with Raven’s Colored Progressive Matrices, Raven, 1995; Portuguese version, Simões, 2000). The groups were significantly different in general language level (HFA: *M* = 83.46, *SD* = 17.22; TD: *M* = 96.89, *SD* = 4.97; assessed with Griffiths Mental Development Scales 2–8 years – Sub-scale Language, GMDS; Luiz et al., 2007), but the difference between groups for receptive vocabulary was non-significant (HFA: *M* = 120.07, *SD* = 34.42; TD: *M* = 142.07, *SD* = 31.51; assessed with Peabody Picture Vocabulary Test, PPVT; Dunn and Dunn, 2007; Vicente et al., 2011, unpublished). All participants were native speakers of European Portuguese, born and raised in monolingual homes in the North of Portugal, with no visual or hearing problems.

**Table 1:** Mean (M), standard deviation (SD) and range for age, non-verbal intelligence, language, and vocabulary of the participants in the high-functioning autism (HFA) and typically developing (TD) groups.

	HFA *(n =* 15)			TD *(n =* 15)			*p-*value^∗^
	*M*	*SD*	Range	*M*	*SD*	Range	
Age	7.40	1.50	6–9	7.53	0.99	6–9	>0.05
Non-verbal intelligence	25.33	5.10	17–32	24.00	4.22	17–32	>0.05
Language	83.46	17.22	40–115	96.89	4.97	93–123	<0.05
Vocabulary	120.07	34.42	53–182	142.07	31.51	99–188	>0.05

*^∗^*p* ≤ 0.05 (one-way ANOVA). Maximum score for non-verbal Intelligence = 36. Score for language: *M* = 100; *SD* = 15. Maximum score for vocabulary = 228.*

### Material

#### Prosody

Participants were evaluated with the European Portuguese version of the Profiling Elements of Prosody in Speech-Communication (PEPS-C; original version: Peppé and McCann, 2003; Portuguese version: Filipe et al., 2017). This test assesses prosodic skills through twelve subtests: six of the subtests address receptive abilities and the other six address expressive abilities. Each subtest comprises 2 example, 2 training, and 16 experimental items. The following is a description of each subtest.

(1) Short-Item Discrimination: assesses the ability to perceive intonation in short-utterances of 1–3 syllables through same/different trials. Two sounds are presented, and the participant indicates whether the sounds are the same or different by clicking on either a symbol for ‘same’ (two red circles) or one for ‘different’ (red circle and green square).
(2) Short-Item Imitation: assesses the ability to imitate intonation in short-utterances of 1–3 syllables. The participant imitates different types of intonation patterns.
(3) Long-Item Discrimination: assesses the ability to perceive prosodic differences in utterances of 3–6 words. The task is the same as Short-Item Discrimination, but with longer stimuli.
(4) Long-Item Imitation: assesses the ability to imitate prosodic differences in utterances of 3–6 words. The participant imitates different types of intonation patterns.
(5) Affect Reception: assesses the ability to understand liking or disliking intonation. A sound stimulus with a liking or disliking intonation is presented simultaneously with a picture of the stimulus. Then two images showing a happy and a sad face appear, and the participant selects the image corresponding to the intonation pattern heard (i.e., happy face for liking intonation or sad face for disliking intonation).
(6) Affect Expression: assesses the ability to produce liking or disliking intonation. The participant produces liking or disliking intonation, and shows what he/she wants to convey with the utterance produced by pointing to the sad or happy face.
(7) Turn-end Reception: assesses the ability to understand question versus statement intonation. The participant hears a declarative or interrogative pattern and identifies the pattern selecting one of two pictures (i.e., the participant chooses the picture of a child offering a food item, when hearing a question; or chooses the picture of a child reading a book showing the object mentioned in the utterance, when hearing a declarative).
(8) Turn-end Expression: assesses the ability to produce question versus statement intonation. One picture of food offered or read out appears on the screen, and the participant says the item with suitable intonation (i.e., interrogative or declarative pattern).
(9) Chunking Reception: assesses the ability to comprehend syntactically ambiguous phrases disambiguated by prosody. The participant hears an auditory stimulus that may correspond to two or three items (e.g., *Fish-Fingers and Fruit* vs. *Fish, Fingers and Fruit*). Then, he/she chooses if the utterance heard matches a picture with two or three items.
(10) Chunking Expression: assesses the ability to produce utterances disambiguated by prosody. Pictures with two or three items (e.g., *Fish-Fingers and Fruit* vs. *Fish, Fingers and Fruit*) are presented and the participant describes what he/she is seeing.
(11) Focus Reception: assesses the ability to identify focus. The participant sees two colors on the screen and hears an utterance with focus (i.e., main stress) in one color (e.g., *Blue and BLACK socks*). Then, he/she points to the color that was focused (e.g., *Black*);
(12) Focus Expression: assesses the ability to produce focus. The participant sees a picture and hears a sentence that does not match the picture (e.g., *The black cow has the ball*). Then, he/she corrects the speaker producing the matching sentence (e.g., *The RED cow has the ball*).

Different reasons led us to prefer the PEPS-C test to other ways of testing prosody: (1) it is a comprehensive prosodic test already used with children with autism; (2) to our knowledge, it is the only prosodic test available for European Portuguese that assess both receptive and expressive prosodic abilities; (3) it does not require specialized transcription skills; and (4) responses are the same for all participants.

#### Executive Functions

Participants were evaluated with the Children’s Color Trails Test (CCTT, Llorente et al., 2003). The CCTT consists of two parts: CCTT-1 and CCTT-2. The CCTT-1 measures visual tracking, processing speed, and graphomotor skills. The CCTT-2 is a more complex task that adds divided attention, set-switching, inhibition, and working memory/sequencing. In both parts, participants connect circled numbers (1–15) with a pencil in ascending order, but in CCTT-2 the numbers alternate in color (pink and yellow). Moreover, the CCTT allows the computation of an Interference Index that measures the added task requirements of CCTT-2 using Time raw scores (raw scores completion time in seconds) through the following formula: (CCTT-2 Time raw score – CCTT-1 Time raw score)/Time CCTT-1 Time raw score.

The CCTT test was chosen to test EFs for the following reasons: (1) it reduces the impact of linguistic components including administration guidelines, because visual instructions allow administration without the linguistic component; and (2) it overcomes limitations of an older similar test, the Children’s Trail Making Test (Reitan, 1971), which uses a combination of the English alphabet with colors, that might exclude children with language or learning disabilities.

### Procedure

Informed consent was obtained from participants’ parents/caregivers, who had the opportunity to ask for further information about the study. Each child was assessed individually in a quiet room with adequate lightning conditions, at their school, in their home, or at the University of Porto.

The assessment was performed in two to three sessions completed within a month and lasting approximately 45 min each. Administration order was the same for all the participants: CCTT-1, CCTT-2, and PEPS-C (Short-Item, Long Item, Turn-End, Affect, Chunking, and Focus). In PEPS-C, half of the participants started with the receptive tasks and the other half with the expressive tasks.

## Results

For the PEPS-C tasks, each participant’s answer was scored as correct (with 1 point) or incorrect (with 0 points). PEPS-C allows the computation of a score for each subtest (maximum = 16), and the computation of a total score that corresponds to the sum of all subtests (maximum = 192). For the CCTT test, scores involved the completion test time in seconds of CCTT-1 and CCTT-2. Additionally, the CCTT Interference Index was computed to measure the added task requirements of CCTT-2.

### HFA and Typically Developing Group Comparisons

To examine performance differences between the HFA and TD groups on the PEPS-C and the CCTT, a comparative analysis was conducted. The results were analyzed separately for each PEPS-C task (Short-Item Discrimination, Short-Item Imitation, Long-Item Discrimination, Long-Item Imitation, Turn-End Reception, Turn-End Expression, Affect Reception, Affect Expression, Chunking Reception, Chunking Expression, Focus Reception, and Focus Expression) and for each CCTT component (CCTT-1, CCTT-2, and CCTT Interference Index; see **Table 2** for details).

**Table 2:** Scores in PEPS-C tasks and CCTT components in the high-functioning autism (HFA) and typically developing (TD) groups.

	TD Group	HFA Group
	Mean (*SD*)	Mean (*SD*)
**PEPS-C**	**155 (14.7)^∗^**	**133 (33.6)^∗^**
**Short-item discrimination**	**15.20 (1.21)^∗^**	**12.47 (3.58)^∗^**
**Short-item imitation**	**14.90 (1.49)^∗^**	**11.50 (3.98)^∗^**
Long-item discrimination	12.80 (1.27)	11.13 (5.30)
Long-item imitation	14.93 (1.16)	12.13 (5.27)
**Turn-end reception**	**15.07 (1.62)**	**13.20 (3.57)^∗^**
**Turn-end expression**	**14.13 (2.72)^∗^**	**11.40 (3.79)^∗^**
Affect reception	15.13 (1.13)	14.27 (2.99)
**Affect expression**	**13.13 (2.92)^∗^**	**8.93 (5.71)^∗^**
Chunking reception	12.73 (2.25)	11.73 (3.08)
Chunking expression	11.40 (2.58)	10.67 (3.73)
Focus reception	10.93 (2.89)	11.73 (2.93)
Focus expression	4.53 (4.08)	3.80 (4.29)
CCTT-1	49.63 (27.24)	57.20 (37.63)
CCTT-2	76.08 (36.50)	132.25 (132.57)
**CCTT interference index**	**0.66 (0.46)^∗^**	**1.29 (0.83)^∗^**

*^∗^*p* < 0.05.*

In the PEPS-C, the difference between groups on the overall mean score was significant: *F*(1,28) = 5.214, *p* = 0.030; η^2^ = 0.157. In all the PEPS-C tasks, HFA children showed lower scores, however the differences were significant only for Short-Item Discrimination [*F*(1,28) = 4.244, *p* = 0.049; η^2^ = 0.132], Short-Item Imitation [*F*(1,28) = 10.975, *p* = 0.003; η^2^ = 0.282), Turn-End Reception (*F*(1,28) = 4.847, *p* = 0.036; η^2^ = 0.148), Turn-End Expression (*F*(1,28) = 4.959, *p* = 0.034; η^2^ = 0.150), and Affect Expression (*F*(1,28) = 6.322, *p* = 0.018; η^2^ = 0.184).

In the CCTT, no difference between groups was found for the time required to complete CCTT-1 or CCTT-2 (*F*(1,28) > 1; *F*(1,28) = 2.503, *p* = 0.125; respectively). However, a significant difference between groups for the CCTT Interference Index was found (*F*(1,28) = 6.710, *p* = 0.015; η^2^ = 0.193; see **Table 2**).

### Correlations Between the EFs Test and the Prosodic Test

**FIGURE 1:**
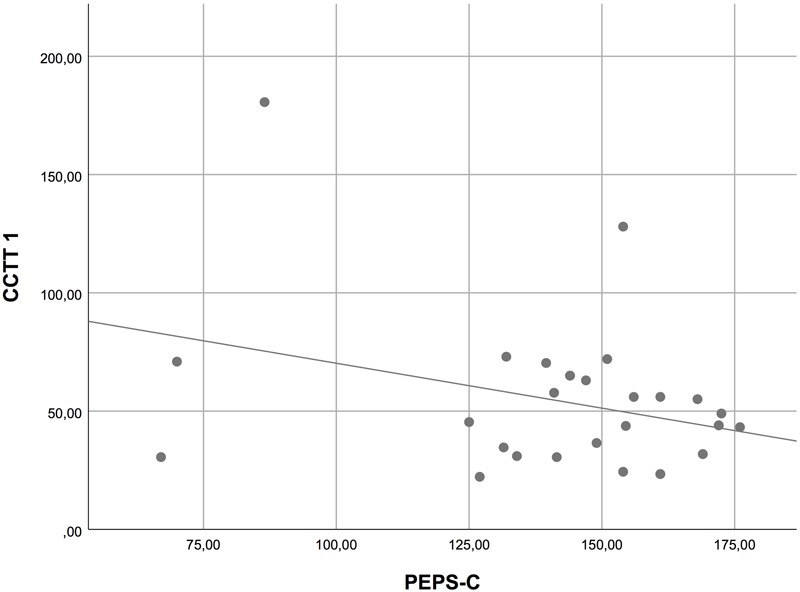
Scatter plot displaying the correlation between PEPS-C and CCTT-1.

**FIGURE 2:**
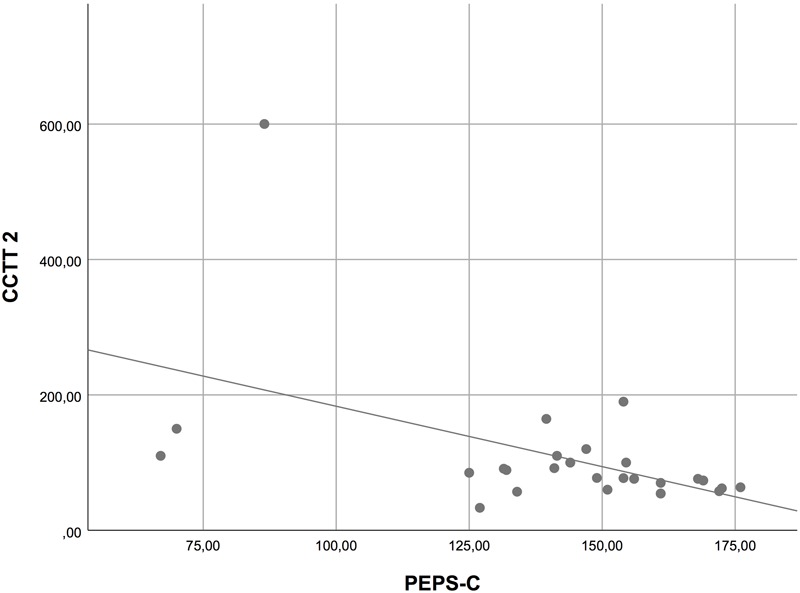
Scatter plot displaying the correlation between PEPS-C and CCTT-2.

**FIGURE 3:**
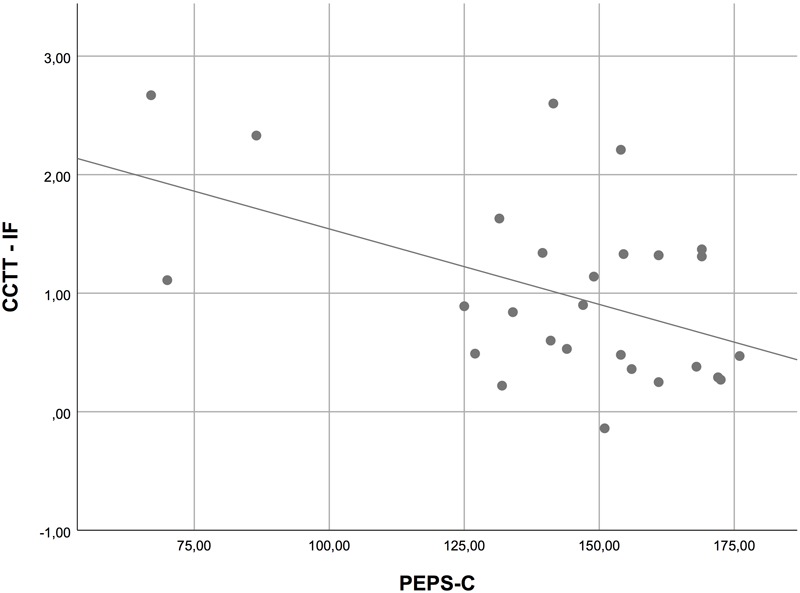
Scatter plot displaying the correlation between PEPS-C and CCTT Interference Index (CCTT-IF).

In order to analyze the relation between possible prosodic impairments and other basic deficits, we computed Pearson correlations between variables. We used the overall mean score of the PEPS-C and the scores in the different components of the CCTT. For both groups together (i.e., HFA and TD children), we found no correlation between PEPS-C and CCTT-1 (see **Figure 1**), but moderate correlations were found between the PEPS-C and CCTT-2 (Pearson’s *r* = 0.50, *p* < 0.001; see **Figure 2**), and between the PEPS-C and the CCTT Interference Index (Pearson’s *r* = 0.48, *p* < 0.001; see **Figure 3**). Additionally, correlations between PEPS-C individual tasks and CCTT components were also calculated, with receptive tasks being more correlated with CCTT components than expressive tasks (the exception is Affect Expression), and CCTT-2 and CCTT Interference Index generally showing stronger correlations with PEPS-C tasks (see **Table 3** for details). However, when the groups were considered separately, the correlations lost statistical significance.

**Table 3:** Correlations between PEPS-C tasks, CCTT-1, CCTT-2, and CCTT interference index.

PEPS-C tasks	CCTT-1	CCTT-2	CCTT interference index
Short-item discrimination	-0.24	-0.47^∗^	-0.60^∗∗^
Short-item imitation	-0.08	-0.13	-0.18
Long-item discrimination	-0.22	-0.31	-0.36^∗^
Long-item imitation	-0.23	-0.38	-0.44^∗^
Turn-end reception	-0.32	-0.48^∗^	-0.35
Turn-end expression	-0.22	-0.45^∗^	-0.40^∗^
Affect reception	-0.53^∗∗^	-0.68^∗∗^	-0.44^∗^
Affect expression	-0.37^∗^	-0.45^∗∗^	-0.25
Chunking reception	-0.32	-0.36^∗^	-0.23
Chunking expression	-0.12	-0.01	-0.18
Focus reception	-0.20	-0.29	-0.31
Focus expression	-0.12	-0.15	-0.18

*^∗^*p* < 0.05. ^∗∗^*p* ≤ 0.001.*

### Mediation Analysis

**FIGURE 4:**
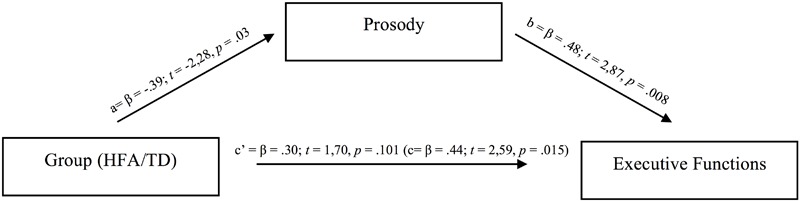
Illustration of mediation analysis (path a = relation between Group and Prosody; path b = relation between Prosody and Executive Functions; path c’ = absence of remaining relation between Group and Executive Functions once Prosody has been added as a mediating factor.

To explore the possible link between EFs (assessed by the CCTT Interference Index) and prosodic impairments in HFA, and to further analyze the group effect, we used a mediation analysis following Baron and Kenny (1986) in the assumption that the effect of an independent variable on a dependent variable is mediated by a mediating variable. First, we examined the hypothesis that prosodic abilities mediate the differences in EFs between the HFA group and the TD group. This hypothesis would be supported if the effect of Prosody (i.e., the mediator) on EFs (i.e., the dependent variable) is greater than the effect of Group (i.e., the independent variable) on EFs, and the effect of Group on EFs is significantly reduced or absent after controlling for prosody. A series of regression analyses were thus conducted to assess (see **Figure 4** for details): (a) the direct effect of Group on Prosody; (b) the direct effect of Prosody on EFs; (c) and the direct effect of Group on EFs.

The direct effect of Group on Prosody (path a in **Figure 4**) showed an adjusted *R*^2^ of.127 (β = -0.39; *t* = -2,28, *p* = 0.03); the direct effect of Prosody on EFs (path b in **Figure 4**) showed an adjusted *R*^2^ of.229 (β = 0.48; *t* = 2,87, *p* = 0.008); and the direct effect of Group on EFs (path c in **Figure 4**) showed an adjusted *R*^2^ of.165 (β = 0.44; *t* = 2,59, *p* = 0.015). All models were significant. However, the effect of Prosody on EFs was larger than the effect of Group on EFs, and the effect of Group on EFs after controlling for Prosody became not significant (path c’ in **Figure 4**; β = 0.30; *t* = 1,70, *p* = 0.101).

Secondly, to analyze the direction of this effect, the reverse regression was performed exploring the effect of Group on Prosody after controlling for EFs. Results showed that Group also became not significant after controlling for EFs (β = 0.23; *t* = -1,26, *p* = 0.218). This suggests that Prosody is mediating differences in EFs between groups, but the reverse pattern also holds.

## Discussion

The relation between EFs and prosody is of interest to researchers and clinicians. The present study extends research on prosodic skills and EFs in autism by investigating these abilities in children with HFA compared to TD peers, and examining the relations between these abilities. Fifteen children with HFA were matched to 15 TD peers on chronological age, gender, and non-verbal intelligence. The PEPS-C was used to assess prosodic performance and the CCTT as an indicator of non-verbal executive control abilities. The results of the present study point to three main findings.

First, HFA children scored significantly lower on the PEPS-C than TD children, pointing to impaired prosodic skills. Lower performance on EFs was also found for HFA children: for CCTT-1 there was no difference between groups; for CCTT-2 the HFA scored worse than TD, although this difference was non-significant; for CCTT Interference Index the difference between groups was significant. These findings show that atypical development affects both prosody and EFs. These results are consistent with findings from other studies reporting that children with HFA performed significantly less well than controls in prosodic tasks (e.g., Rutherford et al., 2002; Peppé et al., 2007) and in EFs tests (e.g., Rajendran and Mitchell, 2007).

Second, the examination of the relation between cognitive processes and prosodic performance in the clinical group showed no correlation between PEPS-C and CCTT-1, but moderate correlations between PEPS-C and CCTT-2, and between PEPS-C and the CCTT Interference Index. Our findings thus suggest no relation between prosodic abilities and visual search and processing speed (assessed by CCTT-1), but a significant association between prosodic deficits and divided attention, working memory/sequencing, set-switching, and inhibition (assessed by CCTT-2 and CCTT Interference Index). Prior research involving children with atypical development also found that deficits in aspects of communication and EFs are associated (e.g., Bishop and Norbury, 2005; Ellis Weismer et al., 2005; Im-Bolter et al., 2006; Henry et al., 2012; Vugs et al., 2014). The current study extends these findings to prosodic abilities and EFs skills.

Third, the results from the mediation analysis showed that the effect of Prosody on EFs was greater than the effect of Group (HFA vs. TD) on EFs, and the effect of Group on EFs after controlling for Prosody became non-significant, thus confirming the hypothesis that prosody influences EFs. The reverse pattern was also found, however, showing that EFs also affect prosodic skills. These results highlight the important (bidirectional) link between EFs skills and prosodic abilities.

Although several studies have described expressive and receptive prosodic impairments in ASD, no consensus has emerged on the characterization of the prosodic profile of this clinical population. From earlier research it is also not clear whether impaired prosody is related to specific cognitive profiles. It is unknown whether deficits in EFs lead to poor communication or whether other cognitive aspects are also at play, influencing the development of EFs and communication (e.g., Bishop et al., 2014). The present study, by focusing on children with HFA, sheds some light on the link between prosodic abilities and EFs, while controlling for the confounding cognitive difficulties related to intellectual disabilities that usually characterize atypical populations. The finding of an association between prosodic impairments (and therefore communication deficits) and EFs in the current study thus presents an important contribution to this research field. Such association is evident not only in the fact that prosodic abilities and EFs are related, but also in the mediating role of prosodic abilities in EFs performance, and vice-versa, with our results pointing to poorer prosodic abilities leading to poorer EFs, and poorer EFs influencing poorer prosodic abilities. Even though our findings did not provide a clear answer about the direction of the relation between EFs and prosodic abilities, as they suggest that the influence is bidirectional, this strong influence raises the important question that shared genetic mechanisms could be involved in the development of both abilities. Bishop et al. (2014) suggest that delayed development of frontal lobes may impact on brain regions that are important for EFs and language processing. Both EF and prosodic abilities emerge early in development, but continue to develop until later ages, with adult-level performance on many tests of EF and prosodic skills being reached at puberty, and performance on many measures continuing to change into adulthood (e.g., Anderson, 2002; Peppé and McCann, 2003; Wells et al., 2004; Best et al., 2009; Filipe et al., 2017). Therefore, the comorbidity between difficulties in EF and prosodic impairments could be a consequence of shared genetic mechanisms.

Childhood communication disorders are associated with different neuropsychological problems. The most commonly associated neuropsychological deficits are problems involving attention and EFs that are usually a common denominator in the different clinical pictures of language disorders. Although the linguistic signs of these disorders are fairly well understood, the associated neuropsychological signs have yet not been studied. It is hoped that the present study is a first step in this direction. As EFs play an important role in the cognitive control of behavior, clinicians, such as speech-language pathologists, should be aware of the relation between cognitive behavior and communicative impairments. Our findings suggest that clinicians responsible for the evaluation of patients with a wide variety of cognitive disorders and language impairments should test both language and EFs in their assessments.

This study has a number of limitations that should be carefully considered. One limitation is the use of the PEPS-C as the only measure of prosodic abilities. The PEPS-C involves the explicit use of prosody, and this makes the tasks easier for children with autism because these individuals tend to not attend to socially relevant information, but might be able to process information when their attention is navigated toward it (Senju, 2012). Future studies should provide another kind of measures for prosodic skills, such as acoustic and phonological analysis for expressive skills and online perception tasks for receptive skills, in order to draw a more comprehensive and accurate view of prosodic deficits in autism. In addition, although the test for EFs was carefully chosen for its non-verbal demands, using the CCTT as the only measure of EFs is certainly a limitation. The fact that the mediation analysis relies only on the CCTT Interference Index is yet another limitation. Future studies should measure other components of EFs to better characterize this multidimensional construct.

Future research should also explore the relation between EFs and prosodic abilities with larger sample sizes and more robust statistical analyses to verify if the present pattern of findings can be replicated. An interesting approach to use in future research is the latent variables approach to capture the link between EFs skills and language domains. Furthermore, some studies have shown that methylphenidate, which produces effects in alertness, combats fatigue, and improves attention, also improves language processing in children (Westby and Watson, 2004; McInnes et al., 2007). Thus further evidence for the possible causal relation between EFs and language domains could be provided by studies addressing whether improving EFs also improves prosodic/language/communicative performance.

## Conclusion

The field of communication impairments and EFs promises to continue as an important area of research concerning the challenging problems of autism. The present study provides important and exciting new directions in the research on prosodic and EFs skills in autism, and may be of considerable interest for clinical practice since EFs and prosodic impairments are characteristic of many neurodevelopmental disorders.

## Author Contributions

MF, SF, and SV contributed to the design of the work. MF prepared the first draft of the manuscript. SF and SV revised it critically for important intellectual content. Final version of the manuscript was approved by all authors.

## Ethics Statement

This study was carried out in accordance with the recommendations of ‘European Union Agency for Fundamental Rights’ with written informed consent from all subjects, following Portuguese regulations. All caregivers gave written informed consent in accordance with the Declaration of Helsinki. The protocol was approved by schools’ boards because there is no review board at the University of Porto or Lisbon when this project was conducted. Participants or caregivers of participants were selected via notices in schools and were informed about: (a) The purpose of the research, expected duration, and procedures; (b) Participants’ rights to decline to participate and to withdraw from the research once it has started, as well as the anticipated consequences of doing so; (c) Factors that may influence their willingness to participate, such as potential discomfort or adverse effects; (d) Any prospective research benefits; (e) Limits of confidentiality, such as data coding, disposal, sharing and archiving.

## Conflict of Interest Statement

The authors declare that the research was conducted in the absence of any commercial or financial relationships that could be construed as a potential conflict of interest.
